# Ethyl 2-[*N*-(2-formyl­phen­yl)benzene­sulfonamido]acetate

**DOI:** 10.1107/S160053680900292X

**Published:** 2009-02-06

**Authors:** S. Ranjith, P. Sugumar, R. Sureshbabu, A. K. Mohanakrishnan, M. N. Ponnuswamy

**Affiliations:** a Centre of Advanced Study in Crystallography and Biophysics, University of Madras, Guindy Campus, Chennai 600 025, India; b Department of Organic Chemistry, University of Madras, Guindy Campus, Chennai 600 025, India

## Abstract

In the mol­ecule of the title compound, C_17_H_17_NO_5_S, the two aromatic rings are oriented at an angle of 30.13 (10)°. The ethyl acetate group assumes an extended conformation. Mol­ecules are linked into *C*(7) chains running along the *a* axis by inter­molecular C—H⋯O hydrogen bonds, and the chains are crosslinked *via* C—H⋯π inter­actions, with the sulfonyl-bound phenyl ring acting as an acceptor.

## Related literature

For the activities of sulfonamides, see: Krishnaiah *et al.* (1995 ▶); Dupont *et al.* (1978 ▶); Sethu Sankar *et al.* (2002 ▶). For related literature, see: Bassindale (1984 ▶).
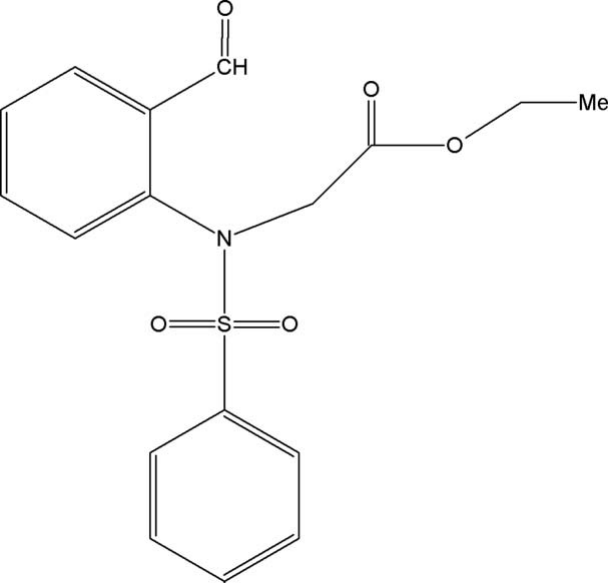


## Experimental

### 

#### Crystal data

C_17_H_17_NO_5_S
*M* *_r_* = 347.38
Orthorhombic, 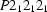

*a* = 11.3512 (6) Å
*b* = 11.7820 (6) Å
*c* = 12.8045 (6) Å
*V* = 1712.47 (15) Å^3^
*Z* = 4
Mo *K*α radiation
μ = 0.22 mm^−1^
*T* = 293 (2) K
0.25 × 0.22 × 0.19 mm

#### Data collection

Bruker APEXII CCD area-detector diffractometer
Absorption correction: multi-scan (*SADABS*; Sheldrick, 1996 ▶) *T*_min_ = 0.948, *T*_max_ = 0.960
13978 measured reflections
5831 independent reflections
3738 reflections with *I* > 2σ(*I*)
*R*_int_ = 0.024

#### Refinement

*R*[*F* ^2^ > 2σ(*F* ^2^)] = 0.042
*wR*(*F* ^2^) = 0.109
*S* = 1.02
5831 reflections
218 parameters
2 restraints
H-atom parameters constrained
Δρ_max_ = 0.29 e Å^−3^
Δρ_min_ = −0.25 e Å^−3^
Absolute structure: Flack (1983 ▶), with 2533 Friedel pairs
Flack parameter: 0.04 (6)

### 

Data collection: *APEX2* (Bruker, 2004 ▶); cell refinement: *SAINT* (Bruker, 2004 ▶); data reduction: *SAINT*; program(s) used to solve structure: *SHELXS97* (Sheldrick, 2008 ▶); program(s) used to refine structure: *SHELXL97* (Sheldrick, 2008 ▶); molecular graphics: *ORTEP-3* (Farrugia, 1997 ▶); software used to prepare material for publication: *SHELXL97* and *PLATON* (Spek, 2003 ▶).

## Supplementary Material

Crystal structure: contains datablocks I, global. DOI: 10.1107/S160053680900292X/ci2739sup1.cif

Structure factors: contains datablocks I. DOI: 10.1107/S160053680900292X/ci2739Isup2.hkl

Additional supplementary materials:  crystallographic information; 3D view; checkCIF report

## Figures and Tables

**Table 1:** Hydrogen-bond geometry (Å, °) *Cg*1 is the centroid of the C8–C13 ring.

*D*—H⋯*A*	*D*—H	H⋯*A*	*D*⋯*A*	*D*—H⋯*A*
C5—H5⋯O4^i^	0.93	2.57	3.220 (2)	127
C16—H16*B*⋯*Cg*1^ii^	0.97	2.75	3.615 (2)	150

Symmetry codes: (i) 
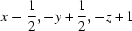
; (ii) 
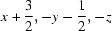
.

**Fig. 1.**
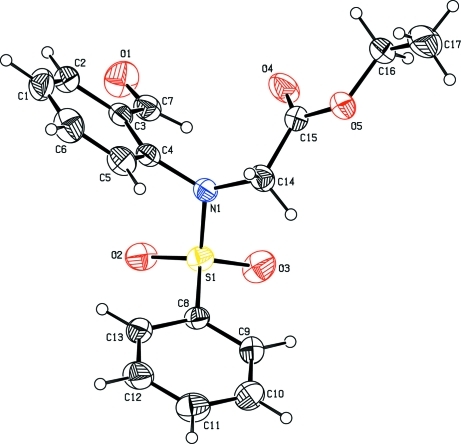
The molecular structure of the title compound. Displacement ellipsoids are drawn at the 30% probability level.

**Fig. 2.**
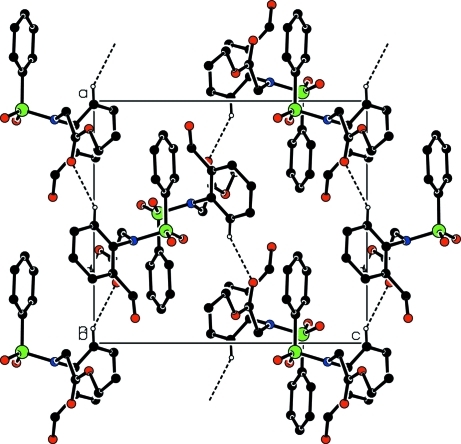
Molecular packing in the title compound, viewed down the b axis. Dashed lines represent hydrogen bonds.

## supplementary crystallographic information

### Comment

The title compound is a potential intermediate for the synthesis of 2-alkylbenzoic acid and exhibits insecticidal, germicidal and antimicrobial activities (Krishnaiah *et al.*, 1995; Dupont *et al.*, 1978). The sulfonamides inhibit the growth of bacterial organism and are also useful for treating urinary and gastrointestinal infections (Sethu Sankar *et al.*, 2002).

Atom S1 has a distorted tetrahedral configuration. The widening of angle O2—S1—O3 [120.46 (10)°] and narrowing of angle C8—S1—N1 [105.97 (8)°] from the ideal tetrahedral value are attributed to the Thorpe-Ingold effect (Bassindale, 1984). The two phenyl rings are oriented at an angle of 30.13 (10)°. The ethylacetate moiety assumes an extended conformation as can be seen from torsion angles C14—C15—O5—C16 of 178.12 (15)° and C15—O5—C16—C17 of 173.12 (19)°.

The molecules are linked into C(7) chains running along the *a* axis by C—H···O hydrogen bonding (Table 1). In addition C—H···π interactions (Table 1) with C8–C13 ring (centroid Cg1) as an acceptor is observed.

### Experimental

2-(Benzenesulfonylamino)benzaldehyde (2 mmol) was added with ethyl bromoacetate (2.2 mmol) in the presence of potassium carbonate (4.7 mmol) and dimethyl acetamide (15 ml). The mixture was stirred at room temperature for 6 h. The reaction mass was poured into crushed ice (50 g) containing 4 to 5 drops of concentrated HCl and extracted with ethyl acetate. The product was obtained by column chromatography (hexane-ethyl acetate 9:1). The removal of the solvent followed by column chromatography of the residue (ethyl acetate) afforded white crystalline solid (yield 25%, m.p. 381-383 K). Single crystals suitable for the X-ray diffraction were obtained by slow evaporation of an ethyl acetate solution of the title compound at room temperature.

### Refinement

H atoms were positioned geometrically (C-H = 0.93-0.97 Å) and were treated as riding on their parent C atoms with U_iso_(H) = 1.2-1.5U_eq_(C). The U^ij^ components of atoms C1, C2 and C6 in the direction of the bond between them were restrained to be equal within an effective standard deviation of 0.001.

### Crystal data

**Table:**

C_17_H_17_NO_5_S	*F*(000) = 728
*M**_r_* = 347.38	*D*_x_ = 1.347 Mg m^−^^3^
Orthorhombic, *P*2_1_2_1_2_1_	Mo *K*α radiation, λ = 0.71073 Å
Hall symbol: P 2ac 2ab	Cell parameters from 5831 reflections
*a* = 11.3512 (6) Å	θ = 2.4–31.9°
*b* = 11.7820 (6) Å	µ = 0.22 mm^−^^1^
*c* = 12.8045 (6) Å	*T* = 293 K
*V* = 1712.47 (15) Å^3^	Block, white
*Z* = 4	0.25 × 0.22 × 0.19 mm

### Data collection

**Table:**

Bruker APEXII CCD area-detector diffractometer	5831 independent reflections
Radiation source: fine-focus sealed tube	3738 reflections with *I* > 2σ(*I*)
graphite	*R*_int_ = 0.024
ω and φ scans	θ_max_ = 31.9°, θ_min_ = 2.4°
Absorption correction: multi-scan (*SADABS*; Sheldrick, 1996)	*h* = −16→16
*T*_min_ = 0.948, *T*_max_ = 0.960	*k* = −17→14
13978 measured reflections	*l* = −19→17

### Refinement

**Table:**

Refinement on *F*^2^	Secondary atom site location: difference Fourier map
Least-squares matrix: full	Hydrogen site location: inferred from neighbouring sites
*R*[*F*^2^ > 2σ(*F*^2^)] = 0.042	H-atom parameters constrained
*wR*(*F*^2^) = 0.109	*w* = 1/[σ^2^(*F*_o_^2^) + (0.0543*P*)^2^ + 0.0353*P*] where *P* = (*F*_o_^2^ + 2*F*_c_^2^)/3
*S* = 1.02	(Δ/σ)_max_ = 0.001
5831 reflections	Δρ_max_ = 0.29 e Å^−^^3^
218 parameters	Δρ_min_ = −0.25 e Å^−^^3^
2 restraints	Absolute structure: Flack (1983), 2533 Friedel pairs
Primary atom site location: structure-invariant direct methods	Flack parameter: 0.04 (6)

### Special details

**Table:**

Geometry. All e.s.d.'s (except the e.s.d. in the dihedral angle between two l.s. planes) are estimated using the full covariance matrix. The cell e.s.d.'s are taken into account individually in the estimation of e.s.d.'s in distances, angles and torsion angles; correlations between e.s.d.'s in cell parameters are only used when they are defined by crystal symmetry. An approximate (isotropic) treatment of cell e.s.d.'s is used for estimating e.s.d.'s involving l.s. planes.
Refinement. Refinement of *F*^2^ against ALL reflections. The weighted *R*-factor *wR* and goodness of fit *S* are based on *F*^2^, conventional *R*-factors *R* are based on *F*, with *F* set to zero for negative *F*^2^. The threshold expression of *F*^2^ > σ(*F*^2^) is used only for calculating *R*-factors(gt) *etc*. and is not relevant to the choice of reflections for refinement. *R*-factors based on *F*^2^ are statistically about twice as large as those based on *F*, and *R*- factors based on ALL data will be even larger.

### Fractional atomic coordinates and isotropic or equivalent isotropic displacement parameters (Å^2^)

**Table:**

	*x*	*y*	*z*	*U*_iso_*/*U*_eq_	
C1	0.6483 (2)	0.49883 (17)	0.57621 (17)	0.0725 (5)	
H1	0.6643	0.5535	0.6266	0.087*	
C2	0.7343 (2)	0.46784 (15)	0.50573 (16)	0.0599 (4)	
H2	0.8076	0.5030	0.5079	0.072*	
C3	0.71222 (15)	0.38413 (13)	0.43117 (13)	0.0455 (4)	
C4	0.60095 (14)	0.33360 (13)	0.42953 (13)	0.0439 (4)	
C5	0.51443 (18)	0.36705 (17)	0.49915 (16)	0.0601 (5)	
H5	0.4401	0.3341	0.4965	0.072*	
C6	0.5388 (2)	0.44896 (19)	0.57199 (17)	0.0749 (5)	
H6	0.4807	0.4711	0.6190	0.090*	
C7	0.80521 (16)	0.35124 (16)	0.35744 (16)	0.0541 (4)	
H7	0.7922	0.2885	0.3149	0.065*	
C8	0.38129 (14)	0.27847 (14)	0.23805 (13)	0.0441 (3)	
C9	0.31891 (18)	0.18498 (16)	0.20531 (16)	0.0567 (5)	
H9	0.3582	0.1211	0.1807	0.068*	
C10	0.1974 (2)	0.18686 (19)	0.20932 (19)	0.0706 (6)	
H10	0.1544	0.1243	0.1867	0.085*	
C11	0.14047 (18)	0.2805 (2)	0.24649 (18)	0.0712 (6)	
H11	0.0586	0.2811	0.2499	0.085*	
C12	0.20264 (19)	0.37386 (19)	0.27891 (17)	0.0684 (6)	
H12	0.1627	0.4371	0.3041	0.082*	
C13	0.32368 (18)	0.37453 (15)	0.27450 (15)	0.0554 (4)	
H13	0.3661	0.4381	0.2955	0.066*	
C14	0.54399 (17)	0.13373 (14)	0.39808 (16)	0.0540 (4)	
H14A	0.5015	0.0911	0.3453	0.065*	
H14B	0.4919	0.1444	0.4573	0.065*	
C15	0.65056 (17)	0.06720 (14)	0.43210 (14)	0.0493 (4)	
C16	0.7056 (2)	−0.10263 (15)	0.51699 (17)	0.0665 (6)	
H16A	0.7630	−0.0624	0.5592	0.080*	
H16B	0.7459	−0.1367	0.4580	0.080*	
C17	0.6456 (3)	−0.1912 (2)	0.5801 (3)	0.1116 (11)	
H17A	0.6075	−0.1565	0.6389	0.167*	
H17B	0.7026	−0.2453	0.6044	0.167*	
H17C	0.5879	−0.2292	0.5378	0.167*	
N1	0.57707 (12)	0.24388 (11)	0.35598 (11)	0.0472 (3)	
O1	0.89698 (13)	0.40067 (14)	0.34903 (16)	0.0853 (5)	
O2	0.58147 (12)	0.38549 (11)	0.21580 (11)	0.0643 (4)	
O3	0.57026 (13)	0.18131 (14)	0.17302 (12)	0.0758 (4)	
O4	0.75012 (13)	0.09354 (12)	0.41701 (15)	0.0768 (5)	
O5	0.61509 (11)	−0.02585 (10)	0.48124 (11)	0.0592 (3)	
S1	0.53632 (4)	0.27483 (4)	0.23629 (3)	0.04953 (12)	

### Atomic displacement parameters (Å^2^)

**Table:**

	*U*^11^	*U*^22^	*U*^33^	*U*^12^	*U*^13^	*U*^23^
C1	0.1104 (14)	0.0522 (10)	0.0549 (11)	0.0040 (10)	−0.0043 (10)	−0.0089 (9)
C2	0.0728 (13)	0.0477 (9)	0.0593 (10)	−0.0020 (9)	−0.0145 (8)	0.0032 (8)
C3	0.0469 (9)	0.0410 (8)	0.0484 (8)	0.0041 (7)	−0.0058 (7)	0.0073 (7)
C4	0.0445 (9)	0.0421 (8)	0.0451 (8)	0.0043 (7)	−0.0039 (7)	0.0069 (7)
C5	0.0544 (11)	0.0629 (11)	0.0629 (11)	0.0072 (9)	0.0080 (9)	0.0063 (9)
C6	0.0906 (13)	0.0731 (12)	0.0611 (11)	0.0163 (12)	0.0180 (13)	−0.0021 (10)
C7	0.0456 (10)	0.0544 (10)	0.0624 (11)	0.0023 (9)	−0.0015 (8)	0.0070 (8)
C8	0.0436 (8)	0.0493 (8)	0.0395 (7)	0.0012 (7)	−0.0038 (6)	−0.0011 (8)
C9	0.0585 (12)	0.0463 (9)	0.0653 (12)	0.0026 (9)	−0.0056 (9)	−0.0044 (8)
C10	0.0562 (12)	0.0676 (12)	0.0879 (15)	−0.0114 (11)	−0.0087 (11)	0.0002 (11)
C11	0.0472 (10)	0.0903 (16)	0.0763 (14)	0.0025 (11)	0.0026 (10)	−0.0014 (13)
C12	0.0615 (12)	0.0784 (14)	0.0653 (12)	0.0238 (11)	−0.0038 (10)	−0.0146 (11)
C13	0.0579 (11)	0.0552 (10)	0.0532 (9)	0.0078 (8)	−0.0074 (8)	−0.0093 (8)
C14	0.0459 (10)	0.0443 (8)	0.0719 (10)	−0.0015 (8)	−0.0038 (9)	0.0079 (8)
C15	0.0518 (10)	0.0414 (8)	0.0546 (9)	−0.0014 (8)	−0.0071 (8)	−0.0024 (8)
C16	0.0828 (15)	0.0493 (9)	0.0674 (12)	0.0114 (10)	−0.0237 (11)	0.0029 (9)
C17	0.133 (3)	0.0727 (16)	0.129 (3)	−0.0176 (16)	−0.048 (2)	0.0442 (17)
N1	0.0434 (7)	0.0416 (7)	0.0567 (8)	−0.0004 (6)	−0.0075 (6)	0.0048 (6)
O1	0.0493 (8)	0.0925 (11)	0.1142 (13)	−0.0147 (8)	0.0139 (8)	−0.0077 (10)
O2	0.0613 (8)	0.0729 (9)	0.0587 (7)	−0.0149 (7)	−0.0028 (6)	0.0174 (7)
O3	0.0589 (9)	0.0942 (11)	0.0743 (9)	0.0085 (8)	0.0091 (7)	−0.0305 (8)
O4	0.0472 (8)	0.0625 (8)	0.1207 (13)	0.0017 (7)	−0.0119 (8)	0.0208 (9)
O5	0.0664 (8)	0.0460 (6)	0.0652 (8)	0.0016 (6)	−0.0078 (6)	0.0095 (6)
S1	0.0434 (2)	0.0569 (2)	0.0483 (2)	0.0004 (2)	0.00160 (19)	−0.00257 (19)

### Geometric parameters (Å, °)

**Table:**

C1—C6	1.376 (3)	C11—C12	1.371 (3)
C1—C2	1.378 (3)	C11—H11	0.93
C1—H1	0.93	C12—C13	1.375 (3)
C2—C3	1.395 (3)	C12—H12	0.93
C2—H2	0.93	C13—H13	0.93
C3—C4	1.396 (2)	C14—N1	1.455 (2)
C3—C7	1.468 (2)	C14—C15	1.506 (3)
C4—C5	1.384 (2)	C14—H14A	0.97
C4—N1	1.442 (2)	C14—H14B	0.97
C5—C6	1.370 (3)	C15—O4	1.188 (2)
C5—H5	0.93	C15—O5	1.327 (2)
C6—H6	0.93	C16—O5	1.443 (2)
C7—O1	1.198 (2)	C16—C17	1.486 (3)
C7—H7	0.93	C16—H16A	0.97
C8—C9	1.375 (2)	C16—H16B	0.97
C8—C13	1.388 (2)	C17—H17A	0.96
C8—S1	1.7605 (16)	C17—H17B	0.96
C9—C10	1.380 (3)	C17—H17C	0.96
C9—H9	0.93	N1—S1	1.6419 (14)
C10—C11	1.365 (3)	O2—S1	1.4253 (13)
C10—H10	0.93	O3—S1	1.4209 (15)
			
C6—C1—C2	120.05 (19)	C13—C12—H12	119.8
C6—C1—H1	120.0	C12—C13—C8	118.67 (18)
C2—C1—H1	120.0	C12—C13—H13	120.7
C1—C2—C3	120.5 (2)	C8—C13—H13	120.7
C1—C2—H2	119.7	N1—C14—C15	111.36 (15)
C3—C2—H2	119.7	N1—C14—H14A	109.4
C2—C3—C4	118.28 (17)	C15—C14—H14A	109.4
C2—C3—C7	119.84 (17)	N1—C14—H14B	109.4
C4—C3—C7	121.88 (16)	C15—C14—H14B	109.4
C5—C4—C3	120.73 (16)	H14A—C14—H14B	108.0
C5—C4—N1	119.76 (16)	O4—C15—O5	125.58 (17)
C3—C4—N1	119.50 (15)	O4—C15—C14	125.54 (16)
C6—C5—C4	119.74 (19)	O5—C15—C14	108.88 (15)
C6—C5—H5	120.1	O5—C16—C17	106.6 (2)
C4—C5—H5	120.1	O5—C16—H16A	110.4
C5—C6—C1	120.6 (2)	C17—C16—H16A	110.4
C5—C6—H6	119.7	O5—C16—H16B	110.4
C1—C6—H6	119.7	C17—C16—H16B	110.4
O1—C7—C3	123.7 (2)	H16A—C16—H16B	108.6
O1—C7—H7	118.2	C16—C17—H17A	109.5
C3—C7—H7	118.2	C16—C17—H17B	109.5
C9—C8—C13	120.88 (16)	H17A—C17—H17B	109.5
C9—C8—S1	119.43 (13)	C16—C17—H17C	109.5
C13—C8—S1	119.68 (14)	H17A—C17—H17C	109.5
C8—C9—C10	119.37 (18)	H17B—C17—H17C	109.5
C8—C9—H9	120.3	C4—N1—C14	117.44 (14)
C10—C9—H9	120.3	C4—N1—S1	119.99 (10)
C11—C10—C9	119.9 (2)	C14—N1—S1	118.12 (12)
C11—C10—H10	120.0	C15—O5—C16	116.89 (15)
C9—C10—H10	120.0	O3—S1—O2	120.46 (10)
C10—C11—C12	120.7 (2)	O3—S1—N1	106.47 (9)
C10—C11—H11	119.7	O2—S1—N1	105.88 (8)
C12—C11—H11	119.7	O3—S1—C8	107.29 (9)
C11—C12—C13	120.43 (19)	O2—S1—C8	109.85 (9)
C11—C12—H12	119.8	N1—S1—C8	105.97 (8)
			
C6—C1—C2—C3	−1.4 (3)	N1—C14—C15—O5	172.45 (14)
C1—C2—C3—C4	0.5 (3)	C5—C4—N1—C14	−59.0 (2)
C1—C2—C3—C7	−179.31 (18)	C3—C4—N1—C14	119.59 (16)
C2—C3—C4—C5	0.9 (2)	C5—C4—N1—S1	96.88 (17)
C7—C3—C4—C5	−179.28 (16)	C3—C4—N1—S1	−84.51 (17)
C2—C3—C4—N1	−177.66 (14)	C15—C14—N1—C4	−81.22 (19)
C7—C3—C4—N1	2.1 (2)	C15—C14—N1—S1	122.42 (14)
C3—C4—C5—C6	−1.4 (3)	O4—C15—O5—C16	−1.6 (3)
N1—C4—C5—C6	177.22 (17)	C14—C15—O5—C16	178.12 (15)
C4—C5—C6—C1	0.4 (3)	C17—C16—O5—C15	173.12 (19)
C2—C1—C6—C5	1.0 (3)	C4—N1—S1—O3	154.32 (14)
C2—C3—C7—O1	−8.5 (3)	C14—N1—S1—O3	−49.95 (15)
C4—C3—C7—O1	171.70 (19)	C4—N1—S1—O2	24.99 (16)
C13—C8—C9—C10	−0.3 (3)	C14—N1—S1—O2	−179.28 (13)
S1—C8—C9—C10	178.31 (17)	C4—N1—S1—C8	−91.66 (14)
C8—C9—C10—C11	−0.6 (3)	C14—N1—S1—C8	64.07 (15)
C9—C10—C11—C12	0.8 (3)	C9—C8—S1—O3	15.45 (17)
C10—C11—C12—C13	0.0 (3)	C13—C8—S1—O3	−165.94 (15)
C11—C12—C13—C8	−0.9 (3)	C9—C8—S1—O2	148.05 (15)
C9—C8—C13—C12	1.0 (3)	C13—C8—S1—O2	−33.34 (17)
S1—C8—C13—C12	−177.55 (16)	C9—C8—S1—N1	−98.00 (15)
N1—C14—C15—O4	−7.8 (3)	C13—C8—S1—N1	80.61 (16)

### Hydrogen-bond geometry (Å, °)

**Table:**

*D*—H···*A*	*D*—H	H···*A*	*D*···*A*	*D*—H···*A*
C5—H5···O4^i^	0.93	2.57	3.220 (2)	127
C16—H16B···Cg1^ii^	0.97	2.75	3.615 (2)	150

Symmetry codes: (i) *x*−1/2, −*y*+1/2, −*z*+1; (ii) *x*+3/2, −*y*−1/2, −*z*.
